# Defects of the Lunacy Law

**Published:** 1924-05

**Authors:** T. C. Mackenzie

**Affiliations:** District Asylum, Inverness


### CORRESPONDENCE.

**[Correspondence on all subjects is invited, but we cannot in any way be responsible for the opinions expressed by our correspondents. No communication can be entertained if the name and address of the correspondent are not given as a guarantee of good faith, but not necessarily for publication. All correspondents should write on one side of the paper only, and we cannot find space for very long letters.]**

### "Defects of the Lunacy Law."

*To the Editor of* The Hospital and Health Review.

Sir,—In your leading article in the April issue of The Hospital and Health Review reference is made to two points specially referred to by Mr. Justice Lush in the course of the case, Harnett *v.* Bond and Adam. Regarding the second point you write as follows: "The other—and much more amazing—fact is that 'when a man is shut up in an asylum, although he may be a perfectly sane man, he is never allowed to know why he is shut up.' The words are those of the judge, who may well have been 'amazed' by such an astonishing discovery."

It appears that Mr. Justice Lush learned in the course of the case that it is not possible or legal for a certified patient in an asylum to be shown or given the medical and other certificates or orders under which he is detained. That, as a broad principle, is true, and the judge was correctly informed as to the fact and expressed his "amazement" at this legal point. It is, however, untrue to say that when a man is shut up in an asylum he is never allowed to know why he is shut up. The contrary is the case. Any patient may be told the reasons for his being detained in an asylum, including facts stated regarding him on his admission papers, and Dr. Bond himself stated, when giving evidence under cross-examination, that no one could be confined in an asylum for years without knowing the reason because it was the practice for doctors to supply the information in conversation with a patient. It seems surprising, in a case where so much is amazing that the remark made by the judge in expressing his amazement should have been considered as equivalent to the statement that a patient could not be shown or given the actual certificates or a copy of them so long as he was detained under treatment. The two things are not the same, but in the minds of many people ignorance, not unassociated in some cases with prejudice, has led to their being considered identical, and this misapprehension tends to be strengthened by the remark, which you quote, of Mr. Justice Lush. T. C. Mackenzie.

District Asylum, Inverness.

